# Association of renal biomarkers with fast progressor phenotype and related outcomes in anterior circulation large vessel occlusion stroke

**DOI:** 10.3389/fneur.2024.1475135

**Published:** 2024-10-30

**Authors:** Lucas Rios Rocha, Mohammad N. Kayyali, Bishow C. Mahat, Abdullah Al-Qudah, Mohamed F. Doheim, Alhamza R. Al-Bayati, Nirav R. Bhatt, Matthew T. Starr, Shlee S. Song, Raul G. Nogueira, Marcelo Rocha

**Affiliations:** ^1^UPMC Stroke Institute and Department of Neurology, University of Pittsburgh School of Medicine, Pittsburgh, PA, United States; ^2^Department of Neurology, Comprehensive Stroke Center, Cedars-Sinai Medical Center, Los Angeles, CA, United States

**Keywords:** ischemic tolerance, large vessel occlusion, anterior circulation, renal function, stroke outcomes

## Abstract

**Background:**

Renal dysfunction is a known predictor of long-term functional dependency after anterior circulation large vessel occlusion (ACLVO) stroke. However, the impact of renal dysfunction on early infarct growth rate (IGR) has not been previously demonstrated. The objective of this study was to define the association of creatinine-based renal biomarkers with fast or slow progressor phenotypes and related clinical outcomes in ACLVO stroke.

**Methods:**

This retrospective study examined patients with acute intracranial internal carotid artery or middle cerebral artery-M1 occlusions admitted between 2014 and 2019. Patients were included if they received baseline CT perfusion (CTP) or MRI on presentation within 24 h of estimated stroke onset. Infarct growth rate (IGR) was determined by ischemic core volume on CTP or MRI divided by time from stroke onset to imaging. IGR was used to stratify fast progressor (IGR ≥10 mL/h) and slow progressor (IGR < 10 mL/h) status. Renal dysfunction was assessed based on serum creatinine and estimated glomerular filtration rate (eGFR) on presenting laboratories. Logistic regression models, adjusted for significant covariates, identified independent associations between renal dysfunction biomarkers, progressor status, and clinical outcomes based on modified Rankin Scale (mRS) at 90 days.

**Results:**

Among 230 patients with ACLVO, 29% were fast progressors, with median serum creatinine levels higher than slow progressors (1.1 vs. 0.9 mg/dL, *p* < 0.05) and lower median eGFR (66.2 vs. 69.0 mL/min/1.73m^2^, p < 0.05). Elevated creatinine (≥1.2 mg/dL) was independently associated with fast progressor status (adjusted OR 2.37, 95% CI 1.18–4.77), worse 90-day mRS (adjusted OR 1.88, 95% CI 1.01–3.51) and mortality (adjusted OR 2.57, 95% CI 1.14–5.79). Reduced eGFR (<60 mL/min/1.73m^2^) was independently associated with fast progressor status (adjusted OR 2.38, 95% CI 1.14–4.94), but not with 90-day mRS or mortality.

**Conclusion:**

Serum creatinine-based biomarkers of renal dysfunction were associated with fast progressor phenotype of ACLVO stroke, and worse clinical outcomes, which may help identify such patients earlier during emergency evaluation for expedited access to EVT. Future prospective studies are warranted to confirm and test implementation of these findings.

## Introduction

The systemic determinants of early infarct growth rate (IGR) and fast versus slow progressor phenotypes in large vessel occlusion (LVO) stroke are incompletely defined. Collateral blood flow is a key predictor of IGR in acute LVO, being modulated by native arteriolar anatomy and hemodynamic factors which vary widely across individuals (1). Age, metabolic syndrome, baseline atherosclerosis, and anemia have also been associated with modulation of collateral capacity and IGR (2–5). Good collateral capacity is strongly associated with slower IGR and improved clinical outcomes (6, 7). Systemic co-morbidities such as hypertension and diabetes are associated with a higher risk of stroke and worse outcomes (8, 9), potentially due to microvascular disease mediated by inflammation and impaired endothelial function (10, 11), representing potential additional mechanisms to early ischemic penumbra loss and core growth in LVO stroke.

Chronic kidney disease (CKD) is an important systemic condition that is prevalent in individuals with hypertension and diabetes due to end-organ damage from microvascular injury (11, 12). Previous research indicates that serum biomarkers of renal dysfunction have also been associated with cerebral microvascular injury and greater incidence of stroke (13, 14). Serum creatinine and estimated glomerular filtration rate (eGFR) are established laboratory indicators of renal insufficiency or prediction of CKD (9, 15). Numerous studies have demonstrated that renal dysfunction, indicated by elevated creatinine and reduced eGFR, is associated with higher stroke severity (16, 17), and worse long-term morbidity and mortality (18). Renal function is often assessed during acute evaluation of stroke patients, particularly those being considered for time-sensitive reperfusion therapies, such as anterior circulation LVO (ACLVO) patients.

Renal dysfunction has been previously associated with cerebral microangiopathy (13), poor collateral flow (19) and higher risk of mortality after endovascular therapy (EVT) for ACLVO (20). This study aimed to determine whether renal dysfunction biomarkers are independently associated with fast or slow progressor phenotypes of ACLVO stroke and related clinical outcomes. Determining whether renal function is associated with early infarct growth during acute ACLVO stroke presentation has the potential to help identify fast progressors on the field and provide novel insight into the pathophysiology of individual tolerance to focal cerebral ischemia.

## Materials and methods

### Population and study design

This was a chart review-based retrospective study of patients with acute intracranial internal carotid artery (ICA) or middle cerebral artery (MCA) M1 segment occlusion who were admitted between 2014 and 2019 at two academic comprehensive stroke centers (Supplementary Figure 1). Patients were included if they underwent baseline magnetic resonance imaging (MRI) or computed tomography perfusion (CTP) on presentation within 24 h after stroke onset, irrespective of subsequent EVT or medical management alone. Patients with missing advanced imaging or serum creatinine data were excluded.

### Data collection and management

Demographics, medical history, National Institutes of Health Stroke Scale (NIHSS) score, basic metabolic laboratories, neuroimaging, treatment, and outcome data were collected by trained stroke researchers. The serum creatinine was obtained upon arrival to the comprehensive stroke center where advanced imaging was performed. The cutoff for abnormally elevated serum creatinine was arbitrarily set at 1.2 mg/dL based on an average threshold for standard female and male laboratory values (15, 21). The creatinine-based eGFR was calculated using the 2021 CKD-EPI equation, which has been extensively validated (22–24). The predictor group for lower glomerular function was defined as eGFR <60 mL/min/1.73m^2^, since it is the main criteria for Stage 3 CKD according to international guidelines (9).

### Progressor phenotypes and key clinical variables

Ischemic core volume was measured using automated RAPID software (iSchemaView; Menlo Park), with thresholds set for CTP (regional cerebral blood flow <30%) or MRI (apparent diffusion coefficient < 620 μm^2^/s). The documented time of last known well was used as a surrogate for time of stroke onset. The IGR was determined by dividing the ischemic core volume (mL) by the time from estimated stroke onset to imaging acquisition (hours). Fast and slow progressor groups were stratified using an IGR cutoff of 10 mL/h as previously determined (6). Patients with IGR ≥10 mL/h were defined as fast progressors, and those with IGR <10 mL/h were defined as slow progressors. Chi-square analysis assessed if witnessed or unwitnessed stroke onset would affect the proportions of fast and slow progressors. The modified Rankin Scale (mRS) was used to measure functional outcomes on discharge and at 90 days post-stroke. Functional independence was defined as mRS 0–2 after stroke. Missing mRS scores at 90 days were imputed from the discharge mRS scores (14% of observations).

### Statistical analysis

All statistical analyses were conducted using STATA 18.5 (StataCorp, College Station, TX). Baseline characteristics between groups were compared using chi-square and Mann–Whitney U tests where appropriate. The model assumptions were verified using the Ramsey RESET test, the Breusch-Pagan test, and the Shapiro–Wilk test, leading to the selection of non-linear models accordingly. Bivariate and multivariate logistic regression modeling was used to identify associations with outcome variables of fast progressor status, functional independence (mRS 0–2) or mortality. Ordinal logistic regression was used for analyses of shift in mRS outcome. Additional regression analyses were conducted to explore the association of BUN-Creatinine ratio greater than 20 (BCr ratio > 20) with progressor status. All multivariate regression models were adjusted for age, sex, NIHSS and covariates with a *p*-value <0.1 in unadjusted group comparisons. An alpha level of 0.05 was considered statistically significant.

## Results

There were 230 patients with acute intracranial ICA or MCA occlusion who met pre-defined inclusion criteria, including 163 (71%) slow progressors and 67 (29%) fast progressors. Demographic, clinical, imaging, and basic metabolic laboratory in the overall population, slow and fast progressor groups are presented in Table 1. In the overall population, the median age was 73 years, 57% were female, 12% were black, and the median body mass index (BMI) was 27.1 kg/m^2^. The median time of last known well to advanced imaging was 6.4 h in the overall cohort, and a sensitivity analysis showed that the prevalence of slow and fast progressors was similar irrespective of witnessed or unwitnessed stroke onset (Supplementary Table 1).

**Table 1:** Baseline patient characteristics per fast and slow progressor status.

Characteristic	All patients *N* = 230	Slow progressor (IGR ≤ 10 mL/h) *N* = 163	Fast progressor (IGR > 10 mL/h) *N* = 67	*p*
Demographics and medical history				
Age, years median (IQR)	73 (62–85)	73 (64–85)	69 (58–81)	0.060
Female Sex N (%)	132 (57%)	94 (57%)	38 (56%)	0.894
Black race N (%)	27 (12%)	18 (11%)	15 (14%)	0.070
Hypertension N (%)	173 (75%)	128 (79%)	45 (67%)	0.908
Diabetes N (%)	64 (28%)	45 (28%)	19 (28%)	0.337
Hyperlipidemia N (%)	104 (45%)	77 (47%)	27 (40%)	0.918
CHF / CAD N (%)	65 (28%)	46 (28%)	19 (28%)	0.878
Atrial Fibrillation N (%)	87 (38%)	62 (38%)	25 (37%)	0.325
Smoking N (%)	50 (22%)	35 (22%)	15 (22%)	0.060
BMI* median (IQR)	27.1 (23.6–31.6)	27.1 (23.4–31.7)	27.0 (23.8–31.5)	0.894
Clinical and imaging profile				
Baseline NIHSS median (IQR)	17 (12–21)	16 (11–20)	20 (15–24)	<0.001
Core volume, mL median (IQR)	14.05 (0–68)	6 (0–24)	99 (41–156)	<0.001
IGR, mL/h median (IQR)	2.60 (0–11.94)	0.74 (0–2.92)	24.26 (14.17–39.53)	<0.001
Basic metabolic laboratories				
Sodium, mM median (IQR)	138 (135–140)	138 (136–140)	137 (135–140)	0.229
Potassium, mM median (IQR)	4.05 (3.7–4.4)	4.00 (3.7–4.3)	4.16 (3.6–4.6)	0.059
Chloride, mM median (IQR)	104 (101–106)	104 (101–106)	103 (99–106)	0.279
Bicarbonate, mM median (IQR)	24.6 (22–27)	24 (22–26)	25 (23–27)	0.069
Glucose*, mg/dL median (IQR)	125 (106–154)	125 (106–154)	125 (107–159)	0.836
BUN, mg/dL median (IQR)	18 (13–24)	18 (14–24)	18 (13–25)	0.674
Creatinine, mg/dL median (IQR)	0.9 (0.8–1.2)	0.9 (0.8–1.1)	1.1 (0.8–1.3)	0.012
Creatinine ≥1.2 mg/dL N (%)	60 (26%)	35 (21%)	25 (37%)	0.013
eGFR, mL/min/1.73m^2^ median (IQR)	67.9 (53.0–86.7)	68.4 (56.2–86.9)	63.8 (44.1–85.0)	0.118
eGFR <60 mL/min/1.73m^2^ N (%)	86 (37%)	55 (34%)	31 (46%)	0.074

CHF/CAD, congestive heart failure and/or coronary artery disease; BMI, Body mass index; NIHSS, National Institutes of Health Stroke Scale; IGR, infarct growth rate; BUN, blood urea nitrogen; eGFR, estimated glomerular filtration rate. *Missing data in three patients for BMI and two patients for Glucose.

Race, diabetes, hyperlipidemia, atrial fibrillation, smoking, and BMI were similarly distributed across fast and slow progressor groups, except for known hypertension which was numerically more prevalent in slow progressors (79%) than fast progressors (67%). In comparison to slow progressors, fast progressors had higher baseline NIHSS (median: 20 vs. 16), larger core volume (median: 99 mL vs. 6 mL) and higher IGR (median: 24.3 mL/h vs. 0.74 mL/h). Serum creatinine was higher in fast progressors (median, 1.1 mg/dL) compared to slow progressors (median, 0.9 mg/dL), with a greater proportion of fast progressors having creatinine levels ≥1.2 (37% vs. 21%, *p* < 0.05). Estimated GFR was numerically lower in fast compared to slow progressors (median: 63.8 vs. 68.4 mL/min/1.73m^2^), with greater prevalence of fast progressors having reduced eGFR (46% vs. 34%, *p* = 0.07). Median potassium and bicarbonate levels were numerically higher in fast progressors (4.2 and 25 mM) than slow progressors (4.0 and 24 mM).

In regression analysis, elevated serum creatinine (≥1.2 mg/dL) was independently associated with fast progressor status (OR 2.18, 95% CI 1.17–4.05, and adjusted OR 2.37, 95% CI 1.18–4.77; Table 2). The association was adjusted for age, sex, hypertension, baseline NIHSS, potassium, and bicarbonate levels. Similarly, reduced eGFR (< 60 mL/min/1.73m^2^) was independently associated with fast progressor status (OR 1.69, 95% CI 0.95–3.02; adjusted OR 2.38, 95% CI 1.14–4.94; Table 3). These data support the independent relationship between creatinine-based markers of renal dysfunction and fast progressor status.

**Table 2:** Association of elevated serum creatinine (≥ 1.2) with fast progressor status.

	OR (95% CI)	*p* value	aOR* (95% CI)	*p* value
Creatinine ≥1.2	2.18 (1.17–4.05)	<0.05	2.37 (1.18–4.77)	0.015
Age	**–**		0.98 (0.95–0.99)	0.034
Sex (Female)	**–**		1.28 (0.66–2.46)	0.464
Hypertension	**–**		0.58 (0.28–1.23)	0.157
Baseline NIHSS	**–**		1.12 (1.06–1.18)	<0.001
Potassium	**–**		1.22 (0.68–2.19)	0.513
Bicarbonate	**–**		1.10 (1.00–1.20)	0.048

*aOR: adjusted OR for co-variables displayed on the table.

**Table 3:** Association of lower glomerular filtration (eGFR <60) with fast progressor status.

	OR (95% CI)	*p* value	aOR* (95% CI)	*p* value
eGFR <60	1.69 (0.95–3.02)	0.077	2.38 (1.14–4.94)	0.020
Age	**–**	**–**	0.97 (0.95–0.99)	0.011
Sex (Female)	**–**	**–**	1.01 (0.53–1.93)	0.982
Hypertension	**–**	**–**	0.56 (0.27–1.19)	0.134
Baseline NIHSS	**–**	**–**	1.12 (1.06–1.18)	0.000
Potassium	**–**	**–**	1.17 (0.64–2.13)	0.607
Bicarbonate	**–**	**–**	1.08 (0.99–1.19)	0.080

*aOR: adjusted OR for co-variables displayed on the table.

In exploratory analysis, we tested whether the observed association between renal dysfunction markers and fast progressor status was driven by pre-renal acute kidney injury which is typically observed with a serum BCr ratio > 20. The analyses (Supplementary Table 2) showed that elevated creatinine remained significantly associated with fast progressor status (adjusted OR 2.33, 95% CI, 1.15–4.69), independently of serum BCr ratio > 20 (OR 0.79, 95% CI, 0.39–1.57). Similarly, eGFR <60 (Supplementary Table 3) remained significantly associated with fast progressor status (adjusted OR 2.32, 95% CI, 1.11–4.84), independently of BCr ratio > 20 (OR 0.80, 95% CI, 0.40–1.59).

**Figure 1:**
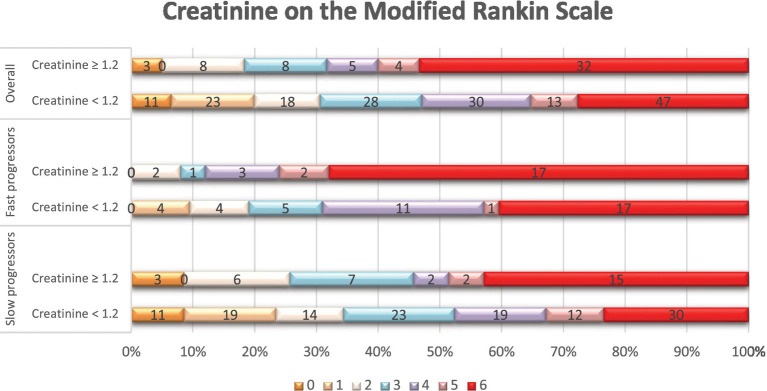
Distribution of scores on the modified Rankin scale at 90 days per serum creatinine status. Shown is the distribution of scores for disability on the modified Rankin scale (mRS) at 90 days among patients with elevated serum creatinine levels (≥ 1.2 mg/dL) and those with normal creatinine levels (< 1.2 mg/dL), in the overall population, fast, and slow progressor strata. The mRS ranges from 0 to 6, with higher scores indicating more severe disability. The numbers in the bars represent the absolute number of patients who had each score; the vertical lines represent percentages at the bottom of the figure.

**Figure 2:**
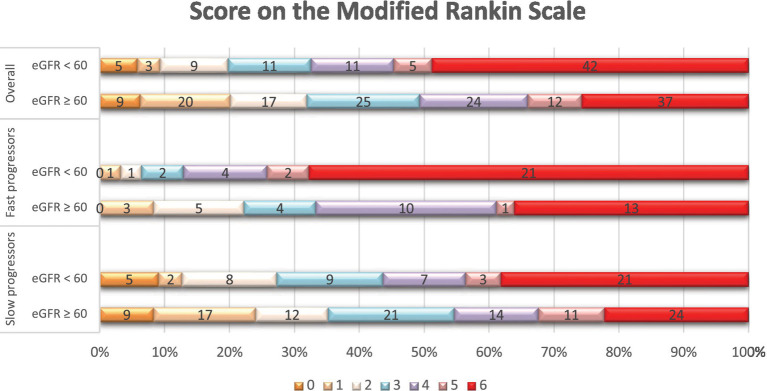
Distribution of scores on the modified Rankin scale at 90 days per eGFR status. Shown is the distribution of scores for disability on the modified Rankin scale (mRS) at 90 days among patients with reduced eGFR (< 60 mL/min/1.73m^2^) and those with normal eGFR (≥ 60 mL/min/1.73m^2^), in the overall population, fast, and slow progressor strata. The mRS ranges from 0 to 6, with higher scores indicating more severe disability. The numbers in the bars represent the absolute number of patients who had each score; the vertical lines represent percentages at the bottom of the figure.

In terms of treatments and outcomes, EVT was performed more frequently in slow progressors (65% vs. 40%, *p* < 0.01), while intravenous tPA was more commonly administered in fast progressors (49% vs. 29%, *p* < 0.01; Supplementary Table 4). At 90 days, fast progressors had a higher mRS score than slow progressors (median: 6 vs. 3, *p* < 0.001). In addition (Table 4), patients with creatinine ≥1.2 had a higher 90-day mRS score than those with creatinine <1.2 (median: 6 vs. 4, *p* < 0.01), and patients with eGFR <60 had a higher mRS score than those with eGFR ≥60 (median: 5 vs. 4, *p* < 0.01). Overall, patients with elevated serum creatinine (Figure 1) or lower eGFR (Figure 2) exhibited a higher proportion of severe disability and mortality compared to those with normal renal function. This trend was similarly observed in both fast and slow progressor groups suggesting an additive effect of renal dysfunction on long-term outcomes. Notably, the proportion of mortality in the fast progressor groups with preserved renal function (creatinine <1.2 or eGFR ≥60) was comparable to the percentage of patients with impaired renal function (creatinine ≥1.2 or eGFR <60) in slow progressor groups.

**Table 4:** Characteristics, treatment and outcome profiles per renal function.

Characteristic	Creatinine < 1.2 *N* = 170	Creatinine ≥ 1.2 *N* = 60	*p*	eGFR ≥ 60 *N* = 146	eGFR < 60 *N* = 84	*p*
Demographics and medical history						
Age, years median (IQR)	73 (62–83)	77 (64–87)	0.218	67 (57–79)	82 (72–87)	<0.001
Female Sex N (%)	104 (61%)	28 (47%)	0.051	72 (50%)	60 (70%)	0.003
Black race N (%)	17 (10%)	10 (17%)	0.168	18 (13%)	9 (10%)	0.643
Hypertension N (%)	125 (74%)	48 (80%)	0.318	98 (68%)	75 (87%)	0.001
Diabetes N (%)	39 (23%)	25 (42%)	0.005	36 (25%)	28 (33%)	0.216
Hyperlipidemia N (%)	71 (42%)	33 (55%)	0.077	59 (41%)	45 (52%)	0.094
CHF / CAD N (%)	42 (25%)	23 (38%)	0.044	32 (22%)	33 (38%)	0.008
Atrial Fibrillation N (%)	59 (35%)	28 (47%)	0.100	44 (31%)	43 (50%)	0.003
Smoking N (%)	39 (23%)	11 (18%)	0.457	41 (28%)	9 (10%)	0.001
BMI* median (IQR)	26.83 (23.6–31.6)	27.4 (23.8–31.7)	0.745	26.8 (23.6–31.6)	27.3 (23.7–32.9)	0.849
Clinical and imaging profile						
Baseline NIHSS median (IQR)	17 (12–21)	19 (15–22)	0.058	16 (12–20)	18 (13–22)	0.045
Core volume, mL median (IQR)	13 (0–64)	27 (4.5–99.5)	0.154	14 (3–71)	15 (0–63)	0.927
IGR, mL/h median (IQR)	2.42 (0–9.86)	4.42 (0.27–22.44)	0.102	2.54 (0.16–10.01)	2.66 (0–16.77)	0.563
Fast Progressors N (%)	42 (25%)	25 (42%)	0.013	36 (25%)	31 (36%)	0.074
Basic metabolic laboratories						
Sodium, mM median (IQR)	138 (136–140)	138 (135–140)	0.799	138 (136–141)	137.4 (135–139)	0.413
Potassium, mM median (IQR)	4 (3.6–4.3)	4.3 (3.9–4.6)	0.004	4.0 (3.6–4.3)	4.2 (3.9–4.5)	0.005
Chloride, mM median (IQR)	104 (101–106)	103 (99.5–106)	0.616	104 (101–106)	104 (99–106)	0.207
Bicarbonate, mM median (IQR)	25 (23–27)	24 (21–26.5)	0.342	24 (22–26)	25 (22–27)	0.500
Glucose*, mg/dL median (IQR)	122 (106–151.5)	132 (107.5–166.5)	0.115	125 (106–155)	125 (106–152)	0.884
BUN, mg/dL median (IQR)	16 (13–21)	25 (21–37)	<0.001	15 (12–19)	24 (20–32)	<0.001
Creatinine, mg/dL median (IQR)	0.9 (0.7–1)	1.4 (1.2–1.6)	<0.001	0.8 (0.7–0.9)	1.2 (1.0–1.5)	<0.001
eGFR, mL/min/1.73m^2^ median (IQR)	76.9 (63.2–90.6)	41.7 (33.2–53.1)	<0.001	80.9 (69.6–95.5)	46.24 (38.1–54.6)	<0.001
Treatment and outcomes						
EVT N (%)	103 (61%)	30 (50%)	0.153	87 (60%)	46 (53%)	0.303
Intravenous tPA N (%)	60 (35%)	20 (33%)	0.784	46 (32%)	34 (40%)	0.242
Symptomatic ICH N (%)	12 (7%)	2 (3%)	0.295	11 (8%)	3 (3%)	0.198
Pre-stroke mRS median (IQR)	0 (0–1)	1 (0–3)	0.022	0 (0–1)	1 (0–2)	0.010
Discharge mRS median (IQR)	4 (3–5)	5 (4–6)	<0.001	4 (3–5)	4 (4–6)	0.001
90-Day mRS* median (IQR)	4 (2–6)	6 (3–6)	0.001	4 (2–6)	5 (3–6)	0.001
Functional independence at 90 days (mRS 0–2) N (%)	52 (31%)	11 (18%)	0.067	46 (32%)	17 (20%)	0.045
Mortality N (%)	47 (28%)	32 (53%)	<0.001	37 (26%)	42 (49%)	<0.001

*Missing data in three patients for BMI and two patients for Glucose. CHF/CAD, congestive heart failure and/or coronary artery disease; BMI, Body mass index; NIHSS, National Institutes of Health Stroke Scale; IGR, infarct growth rate; BUN, blood urea nitrogen; eGFR, estimated glomerular filtration rate, EVT, endovascular thrombectomy, tPA, tissue plasminogen activator, ICH, intracerebral hemorrhage, mRS, modified Rankin Scale.

The independent association of serum creatinine and eGFR with clinical outcomes at 90 days was further evaluated. Overall, patients with either creatinine ≥1.2 or eGFR <60 were also older, had greater prevalence of hypertension, diabetes, hyperlipidemia, heart disease, atrial fibrillation, and smoking. After adjusting for significant demographic and vascular risk factors (Table 5), serum creatinine ≥1.2 mg/dL remained associated with worse mRS at 90 days (OR 2.57, 95% CI 1.47–4.47; adjusted OR 1.88, 95% CI 1.01–3.51). Additionally, elevated creatinine was associated with higher mortality (OR 2.99, 95% CI 1.63–5.50; adjusted OR 2.57, 95% CI 1.14–5.79) but not with functional independence (OR 0.51, 95% CI 0.25–1.06; adjusted OR 0.93, 95% CI 0.36–2.39). Lower glomerular filtration (eGFR <60) was similarly associated with worse ordinal mRS scores at 90 days (OR 2.30, 95% CI 1.40–3.76), although it was not significant after adjustment (adjusted OR 1.09, 95% CI 0.60–1.98). Additionally, patients with eGFR <60 had higher mortality (OR 2.76, 95% CI, 1.57–4.85) and lower functional independence (OR 0.52, 95% CI 0.28–0.99). However, these associations did not reach statistical significance after adjustment for other co-variables (adjusted OR 1.46, 95% CI 0.67–3.17 for mortality; adjusted OR 1.16, 95% CI 0.47–2.88, respectively).

**Table 5:** Association of renal dysfunction markers with clinical outcomes at 90 days.

	OR (95% CI)	*p* value	aOR* (95% CI)	*p* value
Serum Creatinine ≥1.2				
Ordinal mRS	2.57 (1.47–4.47)	0.001	1.88 (1.01–3.51)	0.047
Functional independence	0.51 (0.25–1.06)	0.070	0.93 (0.36–2.39)	0.877
Mortality	2.99 (1.63–5.50)	<0.001	2.57 (1.14–5.79)	0.022
Stage 3 CKD (eGFR <60)				
Ordinal mRS	2.30 (1.40–3.76)	0.001	1.09 (0.60–1.98)	0.771
Functional independence	0.52 (0.28–0.99)	0.047	1.16 (0.47–2.88)	0.751
Mortality	2.76 (1.57–4.85)	<0.001	1.46 (0.67–3.17)	0.336

*All models were adjusted for age, sex, race, baseline NIHSS, progressor status, potassium, pre-stroke mRS, EVT, and IV tPA. Creatinine-based models were also adjusted for history of diabetes, hyperlipidemia, and CHF/CAD; eGFR-based models were also adjusted for history of hypertension, hyperlipidemia, CHF/CAD, atrial fibrillation, and smoking.

## Discussion

The main finding of our study is that creatine-based biomarkers of renal dysfunction are independently associated with the fast progressor phenotype and worse clinical outcomes in acute ACLVO stroke. In the overall study cohort, approximately 1 in 3 ACLVO patients presented with impaired eGFR (< 60 mL/min/1.73 m^2^), similar to data reported in other LVO stroke cohorts (25, 26). The relationship between reduced eGFR and worse clinical outcomes in ACLVO patients after EVT has been previously well demonstrated in a recent meta-analysis of 11 international studies (27). Our data support and extend these earlier findings by indicating a novel association between creatinine-based biomarkers of renal dysfunction with rapid early ischemic core growth during ACLVO stroke, which is a known predictor of worse clinical outcomes in this population (6).

Since emergency point-of-care determination of serum creatinine is commonly performed during acute stroke evaluation before contrast-based CT studies, biomarkers of renal dysfunction could potentially aid in the early identification of ACLVO patients with fast progressor phenotype before advanced imaging is available. Therefore, serum creatinine-based biomarkers may have practical implications in the pre-hospital or primary stroke center when hemodynamic management and time-sensitive transfers of fast progressors directly to the neuro-angiography suite may have significant benefit. Early recognition of fast progressors may also have potential use to help enrollment in future clinical trials of hyper-acute neuroprotection targeting patients with large ischemic core. However, data from our study is limited to serum creatinine levels available at the comprehensive stroke center where advanced imaging was obtained, and future studies examining renal dysfunction in the pre-hospital or primary stroke center setting are needed to confirm these contentions.

Possible explanations for the association between renal dysfunction and faster early IGR include CKD-related white matter microangiopathy (11) and reduced leptomeningeal collateral recruitment during ACLVO stroke (28). Castro et al. (13) also found that acute MCA territory stroke patients with CKD had significant impairment in dynamic cerebral autoregulation and increased burden of white matter hyperintensities relative to non-CKD controls. Therefore, patients with renal dysfunction could be potentially more vulnerable to faster early infarct growth due to impaired cerebral autoregulation causing reduced collateral capacity (19) and lower tolerance to acute ischemia due to baseline white matter disease burden (11). Further investigation is needed to elucidate the pathophysiological mechanisms linking renal dysfunction, early infarct growth rate, and individual ischemic tolerance to ACLVO stroke.

In support of previous reports, our data also demonstrated that lower renal function was associated with higher mortality and worse functional outcomes independently of reperfusion therapies. Notably, patients with markers of renal dysfunction consistently experienced worse outcomes in studies that did not consider baseline fast or slow progressor status (18, 27). In our unadjusted analysis, we found that baseline renal dysfunction markers and fast progressor status may have an incrementally worse effect on functional dependency and mortality after ACLVO stroke (Figures 1, 2). It is possible that renal dysfunction also reduces odds of longer-term stroke recovery due to association with pre-morbid burden of microvascular dysfunction and white matter hyperintensities (11, 28). However, our study was not designed to study these potential mechanisms which will need further investigation.

Age and common vascular risk factors such as hypertension and diabetes are also known predictors of worse functional outcomes after EVT for ACLVO stroke (29). Dawod et al. (12) argued that the presence of CKD may be a biomarker of end-organ vascular injury in stroke populations due to underlying hypertension or diabetes, rather than an independent stroke risk factor. Our study found older age and increased prevalence of hypertension, diabetes, hyperlipidemia, coronary disease, and atrial fibrillation in patients with creatinine, eGFR or both criteria for renal dysfunction on hospital presentation. However, abnormally elevated serum creatine conferred a 2-fold increase in odds of worse mRS at 90 days and a 2.5-fold increase in odds of mortality despite adjustment for age and significant vascular risk factors in our cohort. These findings are consistent with other studies demonstrating that renal dysfunction at the time of ACLVO stroke is an independent predictor of worse long-term functional outcomes via yet poorly defined mechanisms (27).

Other studies have further compared the impact of AKI and CKD on long-term outcomes after EVT. In particular, Fandler-Hofler et al. found that development of AKI during the hospitalization rather than pre-morbid CKD was a significant predictor of unfavorable prognosis after EVT (26). Moreover, acute hypovolemic renal insufficiency measured by a high BUN-creatine ratio has been weakly associated with poor outcomes in ischemic stroke (30, 31). In contrast to these previous reports, our supplementary analysis showed that elevated serum creatinine and reduced eGFR remained associated with fast progressor status after adjustment for BUN-Creatinine ratio > 20 (Supplementary Tables 2, 3). Therefore, we stipulate that the observed association between renal dysfunction markers and early rapid infarct growth in ACLVO stroke is more likely due to intrinsic renal impairment rather than acute prerenal azotemia from hypovolemia. In addition to hypovolemia, contrast induced nephropathy is a common contributor to acute renal insufficiency in stroke patients undergoing EVT (32, 33). Future studies are needed to clarify whether the association of creatine-based markers of renal dysfunction on hospital presentation and fast progressor phenotypes are due to acute, chronic, or acute on chronic nephropathy.

Our study has limitations. First, its retrospective design is intrinsically prone to selection bias. Some ACLVO stroke patients were excluded over the study period due to the absence of advanced imaging or laboratory data for serum creatinine measurement on hospital presentation. Second, we used a pre-specified laboratory cut-off point for serum creatinine of 1.2 mg/dL as an acceptable indicator of impaired renal function as previously determined (15, 21, 32). However, serum creatinine and eGFR can vary according to biological sex, race (23), and BMI, particularly in patients with higher muscle mass (9). To control for these potential confounders, our regression models were adjusted for sex and race, but not for BMI since it was similarly distributed across progressor status, creatine, and eGFR strata. Third, recorded serum creatinine values were only available at a single time point rather than over a prolonged period which would have been necessary to confirm CKD (9) and chart diagnosis of pre-morbid CKD were not available. However, the prevalence of patients with eGFR <60 mL/min/1.73 m^2^ in our population is similar to that from other similar cohorts that used alternative formulas for GFR calculation (27), supporting the validity of our estimates of renal dysfunction during acute stroke presentation.

## Conclusion

This study found that biomarkers of renal dysfunction (serum creatinine ≥1.2 mg/dL or eGFR <60 mL/min/1.73m^2^) were associated with a faster progressor phenotype of ACLVO stroke, and worse clinical outcomes, suggesting that serum creatinine could serve as an adjunct in early identification of higher risk ACLVO patients. These findings have potential implications in early management and prioritized transfer of fast progressor ACLVO patients to EVT, and for enrollment in future clinical trials of bridge neuroprotective therapies. Future prospective studies are required in larger and more diverse populations to confirm and test implementation of these findings.

## Data Availability

The raw data supporting the conclusions of this article will be made available by the authors, without undue reservation.
