# Supplementary material for: Association of renal biomarkers with fast progressor phenotype and related outcomes in anterior circulation large vessel occlusion stroke
Source: Front Neurol. 2024 Oct 30;15:1475135. doi: 10.3389/fneur.2024.1475135 (PMC11557537; doi:10.3389/fneur.2024.1475135)
Supplement: Supplementary file 1 [file Data_Sheet_1.docx]

Supplementary Material

# Supplementary Figure 1: Study flow chart of included and excluded patients


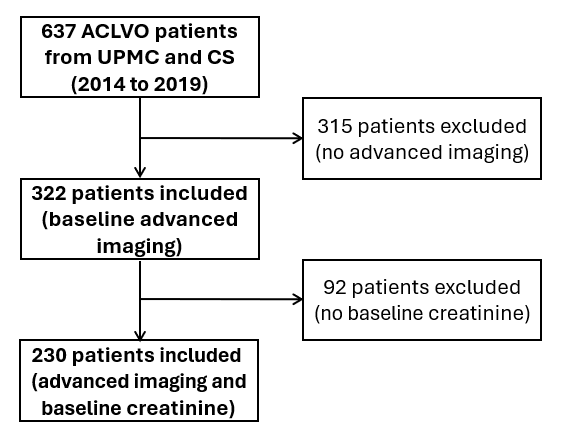


ACLVO = anterior circulation large vessel occlusion; UPMC = University of Pittsburgh Medical Center; CS = Cedars-Sinai Medical Center.

# Supplementary Table 1: Proportions of fast and slow progressors by mode of onset.

|  | **Slow Progressor** | **Fast Progressor** | **Total** |
| --- | --- | --- | --- |
| Wake-up or Unwitnessed | 78 (74%) | 28 (26%) | **106 (100%)** |
| Witnessed | 85 (69%) | 39 (31%) | **124 (100%)** |
| **Total** | *163* | *67* | **230** |

**Chi2** = 0.7022

**P-value** = 0.402

# Supplementary Table 2: Influence of serum BUN-Creatinine ratio greater than 20 (BCr ratio > 20) on the association of elevated serum creatinine (Creatinine ≥ 1.2) with Fast Progressor Status

|  | aOR* (95% CI) | P value | aOR** (95% CI) | P value |
| --- | --- | --- | --- | --- |
| Creatinine ≥1.2 | 2.37 (1.18 - 4.77) | 0.015 | 2.33 (1.15 - 4.69) | 0.018 |
| BCr ratio > 20 | - | - | 0.79 (0.39 - 1.57) | 0.495 |
| Age | 0.98 (0.95 - 0.99) | 0.034 | 0.98 (0.95 - 1.00) | 0.060 |
| Sex (Female) | 1.28 (0.66 - 2.46) | 0.464 | 1.33 (0.68 - 2.58) | 0.404 |
| Hypertension | 0.58 (0.28 - 1.23) | 0.157 | 0.58 (0.28 - 1.23) | 0.157 |
| Baseline NIHSS | 1.12 (1.06 - 1.18) | <0.001 | 1.12 (1.06 - 1.18) | <0.001 |
| Potassium | 1.22 (0.68 - 2.19) | 0.513 | 1.23 (0.68 - 2.22) | 0.498 |
| Bicarbonate | 1.10 (1.00 - 1.20) | 0.048 | 1.09 (1.00 - 1.20) | 0.056 |

*aOR: adjusted OR for co-variables displayed on the table, except BCr > 20.

**aOR: adjusted OR for co-variables displayed on the table, including BCr > 20.

# Supplementary Table 3: Influence of serum BUN-Creatinine ratio greater than 20 (BCr ratio > 20) on the association of lower glomerular filtration (eGFR < 60) with Fast Progressor Status

|  | aOR* (95% CI) | P value | aOR** (95% CI) | P value |
| --- | --- | --- | --- | --- |
| eGFR < 60 | 2.38 (1.14 - 4.94) | 0.020 | 2.32 (1.11 - 4.84) | 0.025 |
| BCr ratio > 20 | - | - | 0.80 (0.40 - 1.59) | 0.520 |
| Age | 0.97 (0.95 - 0.99) | 0.011 | 0.97 (0.95 - 1.00) | 0.021 |
| Sex (Female) | 1.01 (0.53 - 1.93) | 0.982 | 1.05 (0.54 - 2.04) | 0.886 |
| Hypertension | 0.56 (0.27 - 1.19) | 0.134 | 0.56 (0.27 - 1.19) | 0.133 |
| Baseline NIHSS | 1.12 (1.06 - 1.18) | 0.000 | 1.12 (1.06 - 1.18) | 0.000 |
| Potassium | 1.17 (0.64 - 2.13) | 0.607 | 1.18 (0.65 - 2.15) | 0.590 |
| Bicarbonate | 1.08 (0.99 - 1.19) | 0.080 | 1.08 (0.99 - 1.19) | 0.091 |

*aOR: adjusted OR for co-variables displayed on the table, except BCr > 20.

**aOR: adjusted OR for co-variables displayed on the table, including BCr > 20.

# Supplementary Table 4: Treatment and Outcome profile per fast and slow progressor status

| **Characteristic** | **All Patients N = 230** | **Slow progressor (IGR ≤ 10 mL/h) N=163** | **Fast progressor  (IGR > 10 mL/h) N=67** | **P** |
| --- | --- | --- | --- | --- |
| **Treatment and Outcomes** |  |  |  |  |
| Time of LKW to imaging in hours  median (IQR) | 6.4 (3.2-11.8) | 7.9 (3.9-13.9) | 3.7 (1.7-7.4) | p<0.001 |
| EVT  N (%) | 133 (58%) | 106 (65%) | 27 (40%) | p<0.01 |
| Intravenous tPA  N (%) | 80 (35%) | 47 (29%) | 33 (49%) | p<0.01 |
| Symptomatic ICH  N (%) | 14 (6.1%) | 11 (6.75%) | 3 (4.55%) | - |
| Pre-stroke mRS  median (IQR) | 0 [0-2] | 0 [0-2] | 0 [0-2] | - |
| Discharge mRS  median (IQR) | 4 [4-5] | 4 [3-5] | 5 [4-6] | p<0.001 |
| 90-Day mRS*  median (IQR) | 4 [2-6] | 3 [2-6] | 6 [4-6] | p<0.001 |
| Functional independence at 90 days (mRS 0-2)  N (%) | 63 (27%) | 53 (33%) | 10 (15%) | p<0.01 |
| Mortality  N (%) | 79 (34%) | 45 (28%) | 34 (51%) | p<0.001 |

Legend: LKW (last known well), EVT (endovascular thrombectomy), tPA (tissue plasminogen activator), ICH (intracerebral hemorrhage), mRS (modified Rankin Scale).

*Data for thirty-three (14%) patients with a 90-day mRS score were imputed from their discharge mRS data.
